# The Structure of Prejudice and Its Relation to Party Preferences in Belgium: Flanders and Wallonia Compared

**DOI:** 10.5334/pb.335

**Published:** 2017-11-21

**Authors:** Cecil Meeusen, Joris Boonen, Ruth Dassonneville

**Affiliations:** 1 Centre for Sociological Research, KU Leuven, Leuven, BE; 2 Research Center on International Relationship Management, ZUYD Hogeschool, Maastricht, NL; 3 Département de Science Politique, Université de Montréal, Montréal, CA

**Keywords:** Anti-immigrant prejudice, Regional prejudice, Generalized prejudice, Flanders, Wallonia, Propensity to vote

## Abstract

We test two assumptions of the generalized prejudice literature. First, that the structure of generalized prejudice (i.e. how prejudices are interrelated) is dependent on the intergroup context. Second, that different types of prejudice have similar political consequences and run via the generalized prejudice component. We perform these tests in the two main regions of Belgium – Flanders and Wallonia – and investigate the influence of differences in the history of immigration, experience of the linguistic and autonomy conflict, and the separate party system and political discourse (i.e. the societal and intergroup context) on these premises. We make use of the Belgian Election Panel (BEP) data that included measures of prejudice toward multiple target groups (immigrants, Flemings, Walloons, homosexuals, and Jews) and voting propensities for the main political parties. Our results show that, regardless of the differences in intergroup experiences, the structure of prejudice is identical in Flanders and Wallonia. Flemings are, however, more tolerant toward homosexuals and immigrants than Walloons. The political context and the set of potential political outlets does play an important moderating role in the translation of prejudices to party preferences: While negative attitudes toward the other regional group seem to divide the electorate in Flanders, it does not affect voting intentions in Wallonia. Anti-immigrant prejudice is crucial in both regions, but affects voters in different ways at the right-side of the political spectrum.

## Introduction

Although prejudice is specific and directed toward totally different kinds of target groups such as immigrants, homosexuals, and Jews, there is a large consensus that all target-specific prejudices are strongly associated and share a common core labelled “generalized prejudice” (Allport, 1954; Bergh & Akrami, 2016). According to the individual-difference perspective this general tendency to devalue all kind of target groups has its origins in personality traits and cognitive abilities, making it an almost universal phenomenon (Ekehammar, Akrami, Gylje, & Zakrisson, 2004; Hodson & Dhont, 2015). The content of this generalized prejudice, however, is expected to vary between social contexts: “which outgroups become targets of prejudice and discrimination depends on the options a specific society offers” (Zick et al. 2008, p. 367). In other words, while every individual has a predisposition – some a strong one, others a weak one – to think in terms of “us versus them,” how these prejudices are structured and interconnected will be inherent to temporal and contextual constraints. This assumption is the first premise examined in this article.

A second common assumption is that different types of prejudice have similar consequences (Zick et al., 2008). In that sense, observed target-specific consequences can actually be characteristic of a general prejudiced personality, but this goes often unnoticed. A typical example of this phenomenon is the relationship between anti-immigrant prejudice and extreme-right voting. This might be caused by a general devaluation of outgroups, rather than by feelings of prejudice toward this one specific outgroup (i.e. immigrants).

In this article we evaluate these two premises for the Belgian case: (1) whether the structure of generalized prejudice is context-specific, and (2) whether what seem target-specific consequences are actually the result of one’s generalized prejudice predisposition. Belgium is a particularly interesting case in this regard as it consists of two main regions: Dutch-speaking Flanders and French-speaking Wallonia (with bilingual Brussels as a smaller third “capital-region”). The two main regions share important traditions, national identity, and religion, but simultaneously have their own unique intergroup context (Billiet, Maddens, & Frognier, 2006). Moreover, the gradual evolution to more institutional independence between the regions has reinforced a type of regional prejudice and stereotyping that is typical for the Belgian case (Klein, Licata, Van der Linden, Mercy, & Luminet, 2012). If the first premise holds, these regional differences should be reflected in a different configuration of (generalized) prejudice. Therefore, our first goal is to investigate the structure of prejudice in Belgium and to compare this structure between Flanders and Wallonia (RQ 1). To this end, we examine the interrelations between prejudices toward four different target groups (the other regional group, immigrants, Jews, and homosexuals) in the first part of the article.

**Figure 1:**
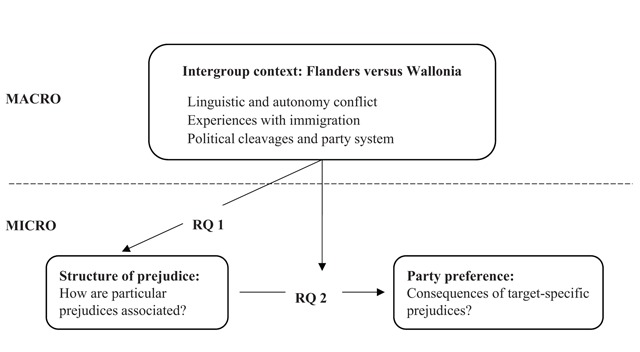
Theoretical model and research questions.

Next, we address the second premise and hypothesize that the particular intergroup context in both regions not only defines how prejudice is structured, but also how prejudice translates into political preferences. We thus investigate how regional intergroup experiences with regard to target groups such as immigrants and the other regional group affect the translation of target-specific prejudices into party preferences in the two main regions of the country (RQ 2). We present both research questions in Figure 1 below.

We aim to contribute to the literature on generalized prejudice in three ways. First, contrary to the dominant personality perspective, we propose a context-based approach to generalized prejudice by comparing the structure of prejudice in two different cultures. Second, including prejudice toward an atypical target group – the other regional group, i.e. Flemings or Walloons – can enhance our understanding of the applicability and boundary of the generalized prejudice concept and its implications for political preferences.^1^ While both are majority groups in their respective region, historical and contemporary political conflicts have activated feelings of threat and hostility toward each other (Klein et al., 2012). Third, studies on the political outcomes of target-specific prejudice disregard the fact that prejudices are highly correlated, missing potential spurious relations. Therefore, we explicitly explore the relation between party preferences and target-specific prejudice, while accounting for the common variance in these target-specific prejudices.

## The structure of prejudice

Different types of prejudice share a strong common component labeled “generalized prejudice” (Allport, 1954; Bergh & Akrami, 2016) or “a syndrome of group-focused enmity” (Zick et al., 2008). Previous research has mainly been directed at discovering the origins of this generalized prejudice component and identified personality traits and ideological factors such as right-wing authoritarianism (RWA) and social dominance orientation (SDO) as crucial predictors (Ekehammar et al., 2004). If generalized prejudice is primarily person-based, it essentially means that this systematic tendency to devalue outgroups can be found within every person, in every society, and at any given time (Zick et al., 2008). Although many authors have confirmed the existence of a one-dimensional generalized prejudice factor capturing prejudice toward a diversity of groups (e.g. Akrami, Ekehammar, & Bergh, 2011; Bierly, 1985; Zick et al., 2008), others stress that some types of prejudice are more similar than other types, resulting in subdimensions or clusters within generalized prejudice (e.g. Bratt, 2005; Duckitt & Sibley, 2007). Depending on group characteristics (e.g. target’s political objectives, intergroup status, or cultural distance) people can react differently to different sorts of groups, resulting in a multidimensional structure of prejudice (Crawford, Mallinas, & Furman, 2015, Meeusen, Meuleman, Abts, & Bergh, 2017). For example, Bratt (2005) found a multidimensional solution for ethnic prejudice in Norway where old immigrant groups formed one cluster and new immigrant groups another one. Nevertheless, these subdimensions of prejudice seem to be highly interrelated and a common generalized prejudice factor can still be distinguished (Beierlein, Kuntz, & Davidov, 2016). Applying these insights to the Belgian context, we expect to find a strong (one- or multidimensional) generalized prejudice structure in both Flanders and Wallonia. This implies that the context-specific “regional prejudice” will be part of the generalized prejudice factor in both regions as well.

***H1:*** *Particular prejudices summarize in a generalized prejudice factor in Flanders and in Wallonia*.

The existence of a generalized prejudice factor, however, does not mean that each kind of target group automatically becomes part of this factor, nor that all types of prejudice are interlinked to the same extent (Meeusen & Kern, 2016). The structure also depends on the socially offered motivations to justify (e.g. perceived threat) or suppress (e.g. social norms) expressions of prejudice toward different target groups (Crandall & Eshleman, 2003; Zick et al., 2008). Particular prejudices that are subject to similar suppression and justification mechanisms will be more strongly connected. Consequently, opportunities in the social and intergroup context (i.e. the interaction between social groups, in a social, economic, political, and cultural environment) will define how prejudices are linked. Therefore, we expect that the structure of prejudice in Flanders and Wallonia will reflect the differences in the intergroup context of these regions, which we describe below (see Figure 1).

Previous research has almost exclusively focused on the origins of generalized prejudice and not on the interpersonal and societal consequences of the structure of prejudice. The generalized prejudice idea assumes that if particular prejudices are highly correlated, they should have similar consequences that find their origin in a general prejudiced personality (Zick et al., 2008). This would imply that previously observed target-specific consequences such as the relation between anti-immigrant prejudice and political preferences for extreme-right parties or discriminatory behavior are characteristic of a broader and general process of outgroup hostility. Therefore, we argue that to get a clear picture of the relationship between prejudices and party preferences, the shared variance between particular prejudices, i.e. the common prejudice component, should be taken into account (for a similar approach see Akrami et al., 2011; Meeusen, Barlow, & Sibley, 2017; Meeusen & Dhont, 2015). Furthermore, we expect the intergroup context of both regions and their separate party systems to moderate the relation between prejudice and party preferences (Figure 1).

We focus on prejudices toward four salient target groups, that each represent a particular prejudice type: Jews as a ethnoreligious minority group, homosexuals as a sexual minority group, immigrants as a common example of an ethnic outgroup, and inhabitants of the other regional group (Flemings or Walloons) as a context-specific group. We put specific emphasis on anti-immigrant and regional prejudice as these two types are idiosyncratic to the particular intergroup context in the two main regions and are expected to be drivers of political behavior. Whereas anti-immigrant prejudice is a well understood phenomenon and has often been linked to voting behavior (Cornelis & Van Hiel, 2015), we argue that in a context of regionalism and an ongoing debate on power redistribution and interregional solidarity, prejudice toward inhabitants of the other region is a central attitude as well. We do not expect structural differences in the position of Jews and homosexuals in both regions (Eeckhout & Paternotte, 2011), nor do we expect these attitudes to play a key role in explaining party preferences (Boonen & Hooghe, 2013). These two target groups are therefore mainly included as a benchmark and allow for testing the idea of generalized prejudice.

## The intergroup context: Flanders versus Wallonia

### Regionalization: Linguistic and autonomy conflict

The evolution toward more regional autonomy or regionalization in Belgium is a process of power conflicts, rooted in seemingly unbridgeable cultural and economic differences between the regions. To accommodate opposing demands of various regionalist movements, the Belgian state has become a double federation of three regions and two main language communities (Deschouwer, 2009): The three regions are Flanders, Wallonia, and Brussels Capital; the two main language groups are Dutch (or Flemish)- and French-speakers.^2^ The issue of power redistribution was initially predominantly focused on linguistic policies and culture-related competences (Deschouwer, 2009, 2013). In Flanders, threat perceptions are based on collective memories of the historical dominance of francophone elites and the related struggle for the protection of the Flemish culture (Billiet, Maddens, & Beerten, 2003; Klein et al., 2012). Among Francophones, feelings of threat stem from this explicit conflict for the preservation of the Dutch language, and from the current dominance of a self-aware, economically leading and unilingual Flanders (Hooghe, 2004). A process of subsequent constitutional reforms from the 1960s onwards has legally formalized and confirmed the pre-existing cultural and linguistic differences in a new, regionalized political structure.

At its origin, the Walloon regionalist movement focused on obtaining economic autonomy as a reaction to the Flemish region’s dominance of the Belgian economy (Swenden & Jans, 2006). Over the past decennia – but particularly from 2007 onwards – the focus of debates between both regions has shifted even more to demands for financial and political autonomy. The Flemish demand for a regional organization of the Belgian social security system, for example, is currently the most visible issue in the power struggle between the regions (Béland & Lecours, 2005; Deschouwer, 2013). This Flemish discourse contrasts with the preference for more interregional solidarity and a durable federal state in Wallonia (Deschouwer, 2013).

The linguistic and cultural differences between Walloons and Flemings, in combination with an ongoing debate on economic independence versus solidarity have provided breeding ground for a distinct type of “regional prejudice,” unique for the Belgian context. Typical stereotypes connected to these conflicts are Flemings as being “intolerant,” “selfish,” and “nationalist”; and Walloons as being “lazy” and “exploiting the social welfare system,” subsided by the “hard-working” Flemings (Klein et al., 2012).

### Experiences with immigration

As other Western European countries, Belgium has a long tradition of immigration. In the aftermath of the Second World War, guest workers – initially from Italy and later from North-African countries and Turkey – were attracted to fill labor shortages. Most non-European immigrants nowadays have Moroccan ancestry (OECD, 2015), which coincides with the public perception about the origins of immigrants (Spruyt, Van der Noll, & Vandenbossche, 2016). Belgians, however, structurally overestimate the percentage of immigrants in the country, enhancing feelings of prejudice toward the group (Hooghe & De Vroome, 2015).

Interestingly, in Flanders and Wallonia immigration is experienced and evaluated differently. These differences seem to reflect contextual characteristics in the region, rather than the characteristics of its inhabitants (Billiet et al., 2006). Wallonia has a longer history of immigration, making Walloons more open toward diversity and the inclusion of newcomers. Walloon integration policies are focused on anti-exclusionism (Martiniello, 2003). However, a deteriorating Walloon economy has activated feelings of competition and threat toward immigration. The situation is somewhat different in Flanders, where a historical struggle for the maintenance of the cultural heritage has generated feelings of cultural threat towards immigrants as well, resulting in restrictive attitudes toward immigration (Billiet et al., 2006).

In Flanders, these feelings of threat are crystalized in the anti-immigrant discourse of a strong extreme-right party, keeping the issue constantly on the political agenda. Right-wing extremism in Flanders also has a strong Flemish identity component and claims the existence of irreconcilable cultural differences between Flemings and Walloons. As such, Flemish nationalism urges thinking in terms of “us” (Flemings) versus “them” (the culturally and economically threatening Walloons and immigrants) and thus combines prejudice toward both target groups in one overarching political ideology (De Wever, 2011). In Wallonia, by contrast, right-wing extremism has never had a real viable political outlet and is not linked to a strong regional identity either (Billiet et al., 2003) (see infra).

In sum, we expect prejudice toward inhabitants of the other region to be more strongly linked to anti-immigrant prejudice in Flanders compared with Wallonia, reflecting its particular intergroup context: In Flanders both target groups are susceptible to similar justification mechanisms such as feelings of cultural and economic threat. Moreover, the presence of a right-wing and Flemish nationalist discourse priming Flemish ingroup identity and targeting Walloons and immigrants simultaneously, further enhances the association between the types. These mechanisms are only marginal or not even present at all in Wallonia.

***H2:*** *Prejudice toward immigrants and prejudice toward the other regional group are more strongly associated in Flanders compared with Wallonia*.

### Political cleavages and separate party systems

The process of regionalization did not only have consequences for power redistribution, but has also affected the structure of the party landscape, which has split along linguistic lines (De Winter, Swyngedouw, & Dumont, 2006). The result is an entirely separated electoral competition: In Flanders only Flemish parties compete and in Wallonia only Francophone parties present lists. This has far-reaching implications for the structure of the political debate and the discourse of political parties, with both party systems focusing on their own regional electorate.

At the level of parties’ discourses there are important differences in the salience of regionalism in both regions. In Flanders, the demand for more regional autonomy is one of the main political cleavages clearly dividing the political landscape. The most obvious proponents are the rightist Flemish nationalist *N-VA* (anno 2016 the largest political formation in Flanders) and the extreme-right *Vlaams Belang*. Explicit opponents of further regionalization are the socialist *Sp.a* and the Green party *Groen* at the left of the political spectrum (Deschouwer et al., 2015). In Wallonia, regional autonomy is far less of a divisive political issue, as all major parties are oriented on the same side in this debate, namely the preference for Belgium as a federal state (Deschouwer & Reuchamps, 2013).^3^ In contrast to the Flemish extreme-right intensely striving for an independent Flanders, the marginal Francophone extreme-right *Démocratie Nationale* (formerly *Front National*) promotes a Belgian Union. The clear mismatch between the territorial demands by a number of major Flemish parties and the reticence of the francophone parties in this respect has led to numerous political crises over the past years (Deschouwer & Reuchamps, 2013).^4^

Studies focusing on this issue have mostly highlighted the link between preferences for (more) regional autonomy and party preferences (Deschouwer et al., 2015), but have not yet focused on the possible underlying negative attitudes toward the inhabitants of the other region. This target-specific regional prejudice is therefore the main focus in our analysis, and can contribute to our understanding of voting intentions in both regions.

As is the case for regionalization, the political issue of immigration has also affected the political debate and party competition in both regions in a different way. In Flanders, anti-immigrant attitudes and the related votes for the extreme-rightist *Vlaams Belang* have extensively been studied (Billiet & De Witte, 2008). This populist Flemish radical right party originated from a dissident party of the Flemish nationalist *Volksunie* in 1978 and its main focus on Flemish independence quickly turned into a populist discourse on immigration (Deschouwer, 2009). The party managed to become the largest party in the 2004 elections for the Flemish Parliament. Although the party also has an explicit discourse striving for Flemish independence and focuses on other issues related to crime and authority, their electorate has based its decision mainly on issues related to immigration (Walgrave & De Swert, 2004). In Wallonia, by contrast, a strong extreme right-wing party has never surged. Furthermore, previous research has indicated that this is not due to a difference at the demand-side, as negative attitudes toward immigrants are equally prevalent in Wallonia (Coffé, 2005). The explanation, hence, seems to lie in the supply-side, as only in Flanders the extreme-right has a well-organized political party with a strong leadership. The Walloon extreme-right party has been visible over the past decades, but thus far has not managed to attract a significant share of voters, making it a marginal electoral player.

In sum, in Flanders feelings of anti-immigrant and anti-Walloon prejudice are explicitly politically mobilized while this is not the case in the Walloon region. We therefore expect anti-immigrant and regional prejudice to be important predictors for party preferences in Flanders; in Wallonia, we expect both types of prejudice to be less relevant for vote choice. Further, we expect the relationships between the prejudice types and party preferences to remain stable when taking into account the generalized prejudice factor, as the political discourse is explicitly framed in terms of the target-group (anti-immigrant, anti-Walloon) and not so much in terms of “us versus anyone else.”

***H3*** *and* ***H4:*** *Prejudice toward immigrants and prejudice toward the other regional group are related to party preferences in Flanders (H3), and this relation remains stable when controlling for generalized prejudice (H4)*.

***H5:*** *Prejudice toward immigrants and prejudice toward the other regional group are not important predictors of party preferences in Wallonia*.

## Data and methods

### Participants

The survey data stem from the Belgian Election Panel 2009–2014, a panel-study on the electoral behavior and public opinion of the Belgian population (Dassonneville, Falk Pedersen, Grieb, & Hooghe, 2014). Participants are 1,542 Belgian residents 18-year and older (*N* = 848 Flemings and *N* = 694 Walloons). Demographically, the Flemish and Walloon sample are comparable with regard to gender (51.4% men in Flemish sample and 50.6% in Walloon sample), education (12.8% higher educated in Flemish sample and 12.3% in Walloon sample), age (Mean of 55.17 years in Flemish sample and 52.24 in Walloon sample), and religion (11% practicing Catholics in Flanders and 9.7% in Wallonia). Unfortunately, the study does not include information on the ethnic background or sexual orientation of respondents. In both regions, women as well as older voters were somewhat underrepresented in the survey compared to the general population. To correct for the underrepresentation of these groups, a sociodemographic weight was applied. This weight ranged between 0.92 and 1.48.

### Procedure and materials

In 2009, a geographically stratified sample of 4,863 voters from the regions of Flanders and Wallonia were selected and contacted for participation. Due to budgetary constraints the Brussels region was not included in the sampling frame, implying that the two main regions of Belgium are focused upon. In 2014, 4,488 citizens of the original 2009 sample were contacted again to participate in a paper-based pre- and post-electoral survey in the context of the 2014 elections in Belgium. In this article, we rely on information of the 2014 pre-electoral survey, as this survey-wave included measures on attitudes toward different target groups. This wave consists of 1,542 completed (and valid) surveys with a response rate of 37.6% among the Flemish and 31.1% among the Walloon respondents. For more information on sampling and representativeness, we refer to the technical report (Dassonneville et al., 2014).

### Measures

To measure different types of prejudice we rely on a range of feeling thermometer scales. Respondents were asked to rate immigrants, homosexuals, Jews, and inhabitants of the other region (Flemings/Walloons) on a scale from 0 = *very negative feeling* to 100 = *very positive feeling*. Admittedly, thermometer ratings capture affective prejudice rather than cognitive forms of prejudice such as beliefs and stereotypes (Tropp & Pettigrew, 2005), but they provide a neutral, evaluative, and content-free measurement making comparisons between groups possible (Bobo & Zubrinsky, 1996).

To investigate the target-specific consequences of prejudice and the impact on party preferences more specifically, we make use of propensity-to-vote (PTV) measures. These measures have been introduced in electoral research by Van der Eijk and his colleagues (2006) and ask voters to indicate on a scale from 0 to 10 their probability to ever vote for each of the parties in the party system. Since their introduction, a large and growing number of studies have relied on such measures to investigate voting intentions, especially in the context of multiparty systems in Europe (De Angelis & Garzia, 2013). The PTV measures are aimed to directly capture respondents’ utility of voting for different parties. Van der Eijk and colleagues (2006) argue that PTVs should be preferred over traditional categorical vote intention or vote choice measures. The reason is that, particularly in multiparty systems such as Belgium, there are usually too few respondents intending to vote for the smaller parties. Particularly when focusing on smaller extreme-right parties, the voters of which are generally underrepresented in election surveys (Swyngedouw, 2001), relying on PTV measures allows for a more reliable analysis of what explains the probability of voting for these parties.

As PTV measures are based on hypothetical questions of ever voting for a party, they are also criticized for having a low level of construct validity. It is argued that voters are not actively and consciously comparing their propensities of voting for different parties. Previous research has thoroughly examined the validity of PTV measures (Van der Eijk et al., 2006). First, this work has shown that a large majority of voters effectively votes for the party they give the highest PTV to. Second, using information on voters’ second choice – measured by means of a vote intention question – these authors have shown that for most voters their second choice is effectively the party receiving the second highest propensity value as well. Finally, analyses of vote choice with, on the one hand a categorical dependent variable, and on the other hand a PTV-measures as the dependent variable, tend to lead to the same substantive conclusions about the determinants of party preferences (Van der Eijk et al., 2006).

For the data at hand we validated the PTV-measures, and found that 94% of the voters in the 2014 pre-electoral survey intended to vote for the party they gave the highest propensity to. We thus feel confident that these measures are valid, and allow for a thorough analysis of predictors of vote choice. For the analyses, we consider all Flemish parties included in the questionnaire.^5^ For the Walloon parties, unfortunately the questionnaire did not include an item of the new party *Parti Populaire*,^6^ all other Walloon parties were included.^7^ It is important to point out that the questionnaire still included an item referring to the *Front National* (*FN*) and not to the many splinter-parties that, at the time of the election, had succeeded this extreme-right party. We assume, however, that supporters of any of its successor parties still indicated a higher PTV score for the *FN*-item. We think this is a valid assumption, as the PTV measures are not gauging the preference of voting in one specific election at stake.

Some typical control variables for research on party preferences in Belgium were included (Deschouwer et al., 2015): age (in years), education (six-point scale, 1 = *no degree*, 6 = *university degree*), gender, socio-economic status (four categories: self-employed, non-manual workers, manual workers, and non-active), and religious practice (four categories: non-religious, Catholic non-practicing, Catholic practicing, and Other). Note that we do not control for other political attitudes or opinions that are likely to correlate with the PTV measures, such as voters’ left-right position, because we do not consider these attitudes to be causally prior to intergroup attitudes.

### Analytic plan

To evaluate whether the structure of prejudice is equivalent in Flanders and Wallonia (RQ 1), we perform multi-group confirmatory factor analysis (Byrne, 2012). We start by fitting a multigroup baseline model with freely estimated parameters to analyze whether generalized prejudice has the same dimensional factor structure in both groups, i.e. Configural Invariance. Second, we assess whether the pair-wise factor loadings of the latent construct (the relationship between the target-specific prejudices and the generalized prejudice component) are equal between the groups, i.e. whether there is Metric Invariance (or Weak Factorial Invariance). Third, we evaluate whether the paired intercepts of the scale items (here mean levels of the target-specific prejudices) are equal across both groups, i.e. whether there is also Scalar Invariance (or Strong Factorial Invariance). For generalized prejudice to have an equivalent meaning in both regions, at least Metric Invariance must be confirmed. Following model fit indices are used to compare the nested models: the Root Mean Square Error of Approximation (RMSEA) which is preferably below .08, the Comparative Fit Index (CFI) and the Tucker-Lewis Index (TLI) preferably above .95, the Chi-square difference test, and the difference between CFI values (preferably lower or equal to .01) (Cheung & Rensvold, 2002; Hu & Bentler, 1999). However, following Little’s advice (2013), when comparing the nested models we evaluate the statistical significance (in terms of model fit) always in combination with its interpretability.

After establishing the structure of prejudice, we evaluate how the target-specific prejudices are related to voting propensities in both regions (RQ 2). Because we have multiple dependent variables – PTVs for each party – we perform a multivariate regression analysis. This way, we reduce type 1 error by estimating all dependent variables in the same model and we take the associations between the voting propensities into account. The latter is important, as PTVs for parties on the left/right will be correlated. Each time, we present a model with and without controls for the generalized prejudice component. To account for missing data and non-normality of certain variables, parameters were estimated with a Full Information Robust Maximum Likelihood Estimator in Mplus 7.3.

## Results

### Structure of prejudice in Flanders and Wallonia

**Figure 2:**
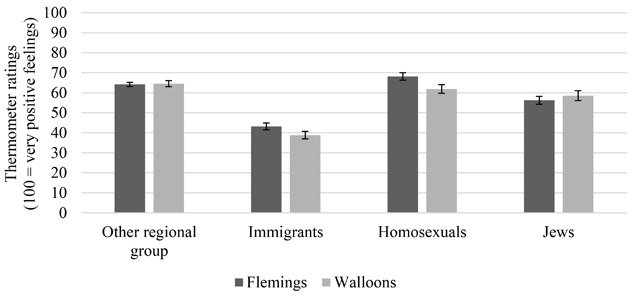
Positive feelings toward different target groups by regional group. *Note*. Bars are 95% confidence intervals around the mean.

Before proceeding with the evaluation of the structure of prejudice, we first look at some descriptive results. Figure 2 shows that Walloons and Flemings agree on the hierarchy in positive feelings toward the four target groups: Homosexuals and the other regional group are liked most, followed by the Jews. Immigrants are disliked most. This hierarchy seems to follow the cultural distance logic: The higher the culturally visible differences with the Belgian majority group, the lower the positive feelings (Hagendoorn, 1995). While Walloons are somewhat more positive toward the Flemings compared to the homosexuals (*p* = .04), Flemings rate homosexuals more positively compared to the Walloons (*p* < .001). Despite the similarity in relative distance, Flemings and Walloons seem to differ in their absolute levels of tolerance. Flemings are more positive toward immigrants (*p* = .003, Cohen’s *d* = .168) and homosexuals (*p* < .001, Cohen’s *d* = .251) compared with Walloons. Both groups are equally positive toward each other (*p* = .787) and the Jews (*p* = .128). These differences in target-specific feelings between Flemings and Walloons remain stable when controlling for education level, SES, gender and age.

All target-specific ratings are positively correlated (Table 1), suggesting a common denominator of “generalized prejudice.” The size of the correlations, however, differs between pairs of prejudices and between the two regional groups. The correlation between feelings toward immigrants and the other regional group is significantly higher (*p* < .001) in Flanders than in Wallonia. As could be expected based on the contextualization of the prejudices, Flemings are indeed more consistent in their evaluation of immigrants and the other regional group compared to Walloons. The relationship between feelings toward Jews and homosexuals is stronger in Wallonia than in Flanders (*p* < .001). Feelings toward homosexuals and immigrants are least correlated in both regions. These differences in size of the correlations remain significant even when controlling for sociodemographic characteristics (i.e., age, education, gender, SES, and religion).

**Table 1:** Correlations between target-specific positive feelings by regional group (Above diagonal = Walloons; Below diagonal = Flemings).

Positive feelings toward…	Other regional group	Immigrants	Homosexuals	Jews

Other regional group	—	**.352**	.399	.469
Immigrants	**.546**	—	.295	.423
Homosexuals	.459	.364	—	**.610**
Jews	.483	.469	**.499**	—

*Note.* All correlations are significant *p* < .001. Correlations in bold are significantly different between the two regional groups.

**Figure 3:**
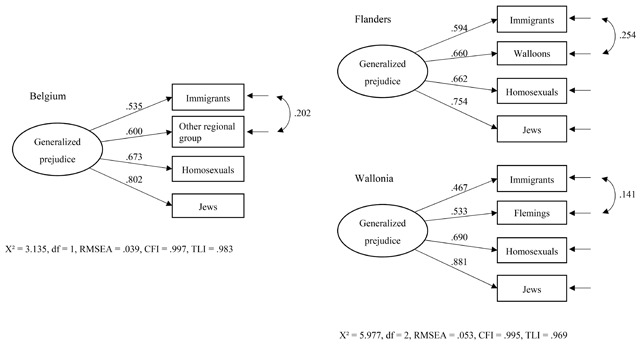
Generalized prejudice structure in Belgium, Flanders, and Wallonia. *Note*. Figure represents Model 1b for Belgium and the Configural Invariance Model 4 for Flanders and Wallonia in Appendix A.

We now formally evaluate the existence of a generalized prejudice structure in Belgium and compare the equivalence of the factor structure in Flanders and Wallonia. We start by estimating a latent generalized prejudice (GP) factor for the whole Belgian sample (Model 1a in Appendix A). The four target-specific prejudices load strongly on the GP factor (loadings between .535 and .802), but modification indices showed that including an error correlation between feelings toward immigrants and the other regional group (*r* = .202) would significantly improve the fit of the model. Next to a mutual overlap between all prejudice indicators captured by the GP factor, feelings toward immigrants and the other regional group have even more in common compared with the other target groups. The model including the error correlation has good fit with the data (Model 1b, χ² = 3.135, df = 1, RMSEA = .039, CFI = .997, TLI = .983) and confirms the existence of a one-dimensional GP factor in Belgium (Figure 3).^8^

Next, we establish a well-fitting baseline model for each region separately (Model 2a to 3b) (Byrne 2012). In Flanders and Wallonia there is considerable overlap between the four target-specific prejudices with an additional error correlation between feelings toward immigrants and Flemings/Walloons. These two models represent the hypothesized multigroup model under test. In a first step, both baseline models are estimated in one model to test whether the number of factors and factor-loading patterns are the same across the regions. This step of Configural Invariance was confirmed (Model 4, χ² = 5.977, df = 2, RMSEA = .053, CFI = .995, TLI = .969): The same one-dimensional factor pattern of GP holds across Flanders and Wallonia (Figure 3). Importantly, this does not mean that the factor structure is identical in both groups. To this end, we have to specify parameter constraints on the pairwise factor loadings, error correlations, and intercepts.

In a second step, we constrained the factor loadings per prejudice type to be equal across groups (Model 5). The comparison of the chi-square, the CFI, and the other fit indices between this model and the configural equivalence model showed that the factor loadings are identical between the groups, hence confirming Metric Invariance.^9^ In a third step, the error correlation between feelings toward immigrants and the other regional group was set to be equal in the two groups (Model 6). Model fit did not worsen which indicates that the error correlation is equivalent among Flemings and Walloons. Finally, also the intercepts per prejudice type were constrained to be equal across groups (Scalar Invariance) (Model 7a). Because restricting all four intercepts significantly deteriorated model fit, we freed the intercept of feelings toward homosexuals (Model 7b) and feelings toward immigrants (Model 7c): Flemings are more positive toward homosexuals and immigrants compared with Walloons. The model fit of this final model was good (χ² = 23.659, df = 7, RMSEA = .058, CFI = .978, TLI = .963). Based on this partial scalar factorial invariance model, latent means of the GP factor can be compared: The levels of GP are not significantly different in Flanders and Wallonia (*p* = .237).

In sum, the analyses show that even though Walloons and Flemings have equal levels of generalized prejudice, they hold different levels of prejudice toward specific target groups: While Jews and the other regional group are equally liked, Flemings are more positive toward immigrants and homosexuals than Walloons. Nevertheless, feelings toward the four target groups can be summarized as one latent generalized prejudice factor and this factor structure is identical in Flanders and Wallonia.

### Prejudice and party preference in Flanders and Wallonia

**Figure 4:**
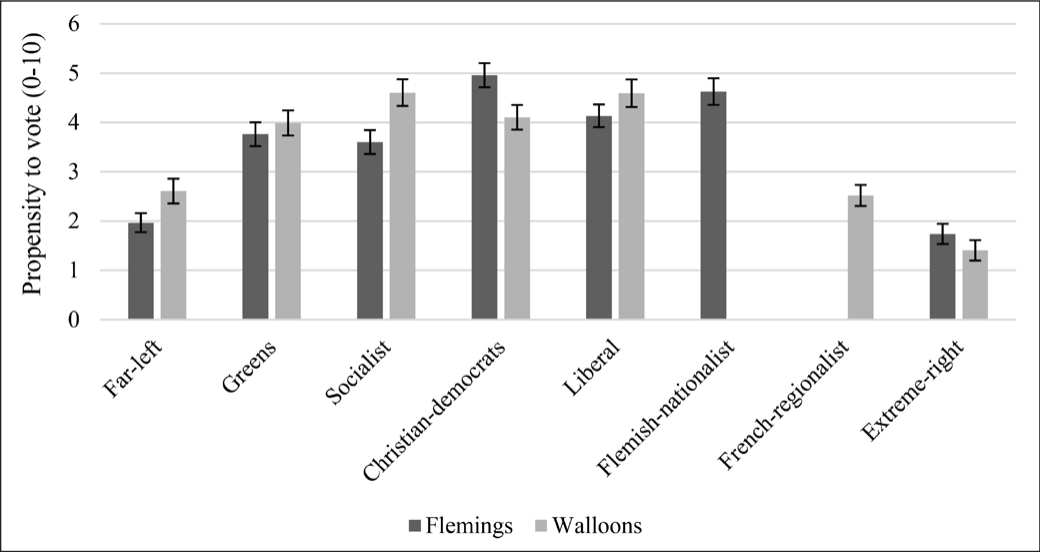
Propensity to vote by regional group. *Note*. Bars are 95% confidence intervals around the mean and can be compared within regions.

In a next step, we examine the target-specific political consequence of prejudice in the two main regions of Belgium by connecting them to party preferences. Before doing so, we offer some descriptives of the dependent variables, the PTV measures for different parties. As evident from Figure 4, Walloon respondents indicate that they have the highest PTV for the Liberal and Socialist party. Flemish respondents, by contrast, are mostly attracted by the Christian-Democrats and the Flemish-nationalist party. In both regions, citizens are least likely to vote for the far-left and extreme-right parties (and the regionalist party in Wallonia).

**Figure 5:**
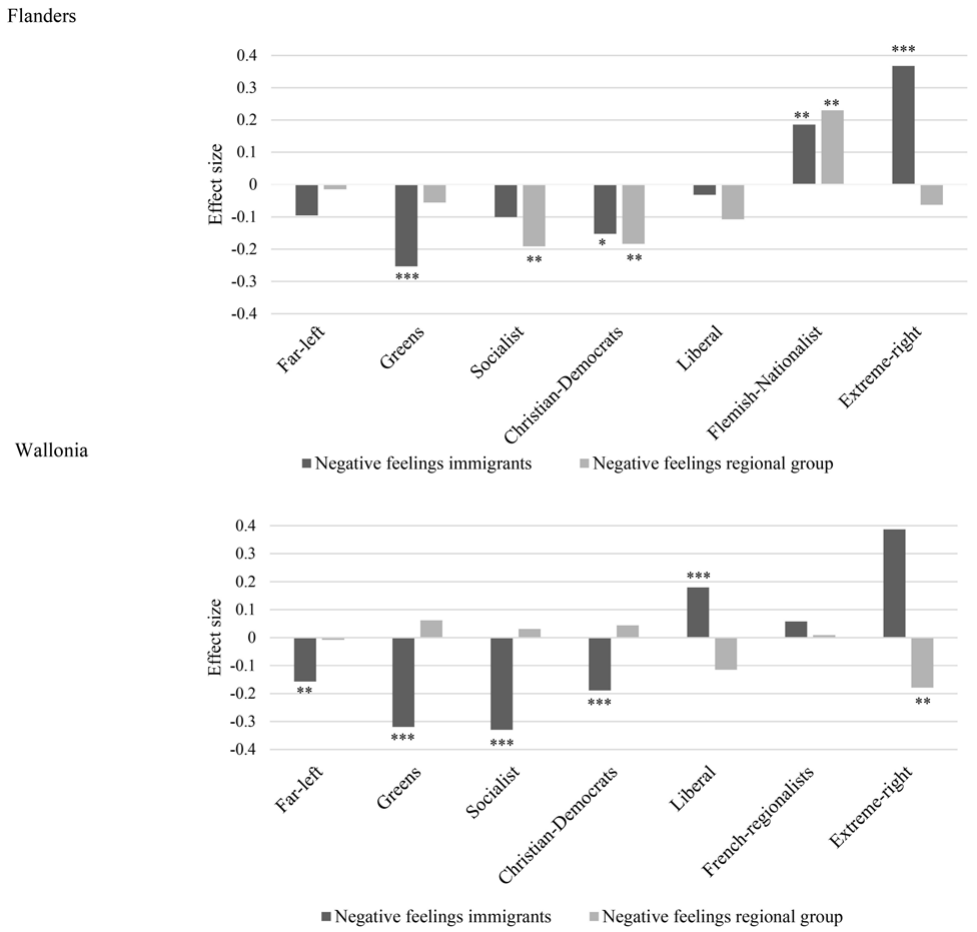
Effect size of relation between target-specific negative feelings and PTVs. *Note*. Effect sizes based on multivariate regression model, controlling for generalized prejudice, age, gender, education, religious practice and SES. Full models in Appendix B. * *p* < .05, ** *p* < .01, *** *p* < .001.

For analyzing the relation between PTVs and feelings toward immigrants on the one hand and the other regional group on the other hand, we proceed in two steps. First, we estimate a model with one target-specific thermometer rating as the independent and the PTVs as the dependent variables. Second, we estimate the same model but control for the generalized prejudice component, i.e. the variance that the four prejudice types have in common. The generalized prejudice component was included as a latent factor of all four target-specific ratings. This way, we correct for the potential spurious effect that target-specific relations are actually due to a general tendency to dislike any kind of group, no matter the characteristics of the target. If the target-specific effect remains significant when the generalized component is taken into account, this means that the relationship between prejudice and party preferences is indeed target-specific and not solely due to a general prejudiced personality. The effect sizes of the relation between PTVs and the target-specific ratings, controlled for the generalized component are presented in Figure 5. The coefficients for the full models, including all control variables can be found in Appendix B.

In Flanders, the immigrant ratings are significantly related to PTVs across all parties. Except for the far-left, liberal, and socialist party, these relationships remain significant when controlling for the generalized component, meaning that the observed relation is not driven by a generalized tendency to (dis)like outgroups, but by the immigrant component in the prejudice type. Flemish respondents with negative feelings toward immigrants are more likely to ever vote for the extreme-right party and the Flemish-nationalist party, but the effect of the latter (β = .186) is only half the size of the former (β = .368). On the other side of the political spectrum, the Green party attracts respondents with less negative feelings toward immigrants (β = –.253). A small negative effect also remains for the Christian-democrats (β = –.152).

Initially, feelings toward the other regional group (here Walloons) are related to the PTV for all Flemish parties. However, when controlling for the generalized component, the association disappears for the far-left, green, liberal, and extreme-right party: Specific feelings toward the Walloons do not predict the PTV for these parties. Nevertheless, the likelihood to vote for the socialists and Christian-democrats is negatively related to the target-specific component of Walloon prejudice (β = –.191 and β = –.183 respectively). As predicted, finally, Flemish respondents with negative feelings toward the Walloons are more likely to vote for the Flemish-nationalist party (β = .230). Surprisingly, however, while a Flemish identity is at the core of extreme-right in Flanders, anti-Walloon attitudes are not related to the PTV of this party once the GP component is accounted for.

In Wallonia we get a slightly different picture. While anti-immigrant feelings are significantly and strongly related to voting propensities once the generalized component is accounted for, this is not the case for negative feelings toward the other regional group (here Flemings). Walloon respondents with negative feelings toward immigrants have, as hypothesized, a higher PTV for the extreme-right (β = .387), but also a higher PTV for the liberals (β = .180). All other parties attract Walloons with a more positive attitude toward immigrants (β = –.157, β = –.320, β = –.330 and β = –.189, for the far-left, greens, socialists and Christian-democrats respectively). Regarding feelings toward the Flemings, we can be brief: There is almost no relationship with the propensity to vote for any Walloon party, except for the extreme-right, which attracts Walloon voters with a positive attitude toward Flemings (β = –.179), reflected by its Belgian nationalist focus. Prejudice is not a decisive motivation to vote for the regionalist party in Wallonia.

The generalized prejudice component is only marginally related to voting propensities. There are some indications that generalized prejudiced respondents are less likely to ever vote for the green and socialist parties and have a higher propensity to vote for extreme-right. These relationships disappear, however, when combined with the anti-immigrant rating. From this, we can conclude that voting propensities are mainly predicted by target-specific anti-immigrant feelings in both Wallonia and Flanders and that feelings toward the regional group are only relevant in Flanders, pointing to an asymmetrical translation of the structure of prejudice in voting propensities.^10^

Finally, the relationships with the control variables follow the patterns that are often observed in similar studies on Belgian voters: The higher educated turn to the green and liberal party, while the lower educated have a higher PTV for extreme-right (and far-left in Flanders). Christian-democrats attract religious voters and liberals the self-employed. Younger respondent are more likely to ever vote for extreme-right and the far-left than older respondents.

## Discussion

We now return to the two central topics of this article – the structure of prejudice in Flanders and Wallonia, and its relation to party preferences – and summarize the main conclusions. First, with regard to the configuration of generalized prejudice, the regional intergroup context is not a key factor. This is shown by our first set of analyses, in which we demonstrate that a one-dimensional generalized prejudice factor indeed exists in Flanders and Wallonia (confirming H1), but that contrary to our expectation (H2), this factor has an identical structure in both regions. Importantly, prejudice toward the other regional group – an atypical context-specific prejudice type – is also part of the central structure of this construct and thus not an exceptional prejudice phenomenon. The fact that generalized prejudice is similarly structured in both regions does, however, not imply that Flemings and Walloons are equally prejudiced toward the target groups. While Flemings are often portrayed as intolerant and selfish (Klein et al., 2012), they have more positive attitudes toward immigrants and homosexuals than their Francophone compatriots. While generally attitudes toward homosexuals, Jews, and the other regional group were relatively positive, prejudice toward immigrants remains a key issue in Belgian intergroup relations.

An important qualification needs to be made. Next to this mutual overlap in all target-specific prejudice indicators, there is an additional association between prejudice toward immigrants and the inhabitants of the other regional group (Flemings or Walloons) in both regions. One explanation for this strong target-specific connection is that prejudice toward both groups has its origins in similar justification mechanisms such as cultural and economic threat (Crandall & Eshleman 2003). In Flanders, contrary to what holds in Wallonia, both the struggle for more regional autonomy and a more stringent attitude toward immigrants are associated with right-wing politics. Therefore, we expected to find a stronger association between the two prejudice types in Flanders than what holds for Walloon respondents. While the error correlation was indeed somewhat more outspoken in Flanders, the difference was not significant. As a result, we found no confirmation for the hypothesized stronger link between anti-immigrant and anti-Walloon prejudice in Flanders (H2).

Further, while we found a one-dimensional solution for the structure of prejudice with this particular set of target groups in Belgium in this study, this does not mean that a multidimensional structure can be excluded. As was discussed, some authors find stronger relations between some target groups, representing subdimensions of prejudice (e.g. Duckitt & Sibley, 2007; Meeusen et al., 2017). Here, we included only four target-groups, a decision that could have influenced the likelihood of finding a one-dimensional solution. Therefore, future research on the structure of prejudice should try to incorporate as many outgroups as possible. Nevertheless, we are convinced that regardless the number of subdimensions, a higher-order generalized prejudice dimension will always appear because this tendency to think in terms of “us versus them” is said to be present in every individual (although in different gradients) (Adorno, Frenkel-Brunswik, Levinson, & Sanford, 1950).

A second main conclusion is that there is a clear target-specific link between feelings toward immigrants and the regional outgroup, and intended political behavior. Interestingly, for anti-immigrant prejudice this link remains present even when we control for the generalized prejudice factor, suggesting that the relation between the structure of prejudice and party preferences is target-specific rather than generalizable across target groups (confirming H4). This is an important finding, as it contradicts the generalized prejudice idea, where it is claimed that all prejudice types should have similar consequences that run via the common prejudice component (Zick et al., 2008). The relation between regional prejudice and some PTVs disappeared when controlling for the GP component. Clearly, anti-immigrant prejudice matters more for voting intentions than attitudes toward Walloons and Flemings. But again, the intergroup context and the range of potential political outlets matters in this respect, as we found some interesting differences between this connection in Flanders and Wallonia.

Not surprisingly, we found that a preference for extreme-right parties is related to anti-immigrant attitudes. More remarkable is how the observed link between the target-specific prejudice types and the PTVs differs in both party systems. In Flanders, it seems that voters with a prejudice toward immigrants and toward the other regional group find their way to the Flemish nationalist party (*N-VA*). This rightist government party indeed pursues a rather firm policy toward immigrants, but its main goal remains realizing Flemish independence. In that sense, the significant link between negative feelings toward Walloons and a PTV for the *N-VA* is not surprising. What is somewhat remarkable is that PTVs for the *N-VA* are also correlated with prejudice toward immigrants. It seems that the *N-VA* serves as a “democratic alternative” for voters with anti-immigrant attitudes who do not have the intention to vote for the less socially acceptable extreme-right party *Vlaams Belang*. The explicit anti-immigrant discourse of this party and its racist reputation might activate a social norm against prejudice in the voter’s mind, making them less likely to express anti-minority political choices as they believe this vote is socially unacceptable (Blinder, Ford, & Ivarsflaten, 2013). Therefore, voters might refrain from voting for the extreme-right party and choose a more socially acceptable option instead: the Flemish-nationalist party. It must be remembered, however, that the effect size of the relationship between anti-immigrant prejudice and voting for the Flemish nationalist party was only half the size of the effect size of the relationship with the extreme-rightist party.

Whereas in the Flemish party system a strong nationalist party as well as a visible extreme-right party are present, these are not (viable) options in Wallonia, and this has consequences for how voters with an anti-immigrant disposition behave. In Wallonia, these voters seem to end up with the liberal party (*MR*), still to the right of the political spectrum. Interestingly, the liberal party in Flanders (that originated from the same traditional national Liberal party as the *MR*) does not attract these prejudiced voters. This creates an interesting paradox for both liberal parties: They attract prejudiced voters in Wallonia – as they are the only main democratic alternative to the right – while they do not attract these voters in Flanders, probably because of the existence of the Flemish-nationalist party. The supply side seems to be of a crucial importance in this respect. Prejudiced voters prefer the option that is situated most to the right, implying that when there are few viable options at this side of the political spectrum, the most right-wing option might just as well be a center party. We should note that there is no immediate reason to assume why a well-organized far-right party in Wallonia could not attract these prejudiced voters (as it did in Flanders). Looking at the trends in the saliency of immigration issues, and the overall scores on anti-immigrant prejudice, Flanders and Wallonia are very comparable. The main difference between both regions should thus mainly be identified at the supply side, and not at the demand side of the voters’ preferences.

Additionally, our results indicate important differences in the salience of anti-regional prejudice in both party systems. Whereas this attitude seems to divide the electorate in Flanders to some extent, it has no predictive power in explaining voting propensities in Wallonia, once the generalized prejudice component is taken into account. Feelings toward immigrants, on the other hand, seem to be an important determinant of intended political behavior in both regions, even more so in Wallonia, so that H3 and H5 are only partly confirmed. These feelings explain preferences on the left-hand side (positive feelings) and on the right-hand side (negative feelings). Once more, differences in the supply-side seem the most obvious explanation for this difference. As there is no viable nationalist party in Wallonia, anti-regional prejudice can simply not be translated in a particular type of political behavior.

A first limitation of the study is the fact that we could not control for additional important factors situated in the field of personality psychology (such as SDO and RWA) and sociology (such as neighborhood characteristics). These factors are important covariates of generalized prejudice and its translation to political behavior (Rink, Phalet, & Swyngedouw, 2009). Similarly, we did not have appropriate measures for ingroup attitudes, which could potentially have affected party preferences as well. Especially with regard to extreme-right voting not including ingroup evaluations might bias the strength of the prejudice relations. Nevertheless, research has shown that outgroup attitudes are still a more powerful predictor of extreme-right voting than ingroup feelings (Billiet & De Witte, 1995; Meuleman & Lubbers, 2013). In an additional analysis we have included attitudes toward the own regional group as a proxy measure for ingroup attitudes in the analyses. The results show that the effect sizes of the prejudice indicators were hardly affected. It must be noted, however, that for a fair number of respondents, the own regional group is probably not the group they identify with most, and therefore, our proxy is not a good ingroup indicator. Second, we did not have data on the Brussels Capital region, which would have given important additional insights in the structure of prejudice and party preferences. In this region, Dutch- and French-speaking citizens live together, which probably affects how they think about each other. Furthermore, in Brussels there is a viable regionalist party, *DéFi*, propagating a strong Walloon identity. In other words, in Brussels Capital there is a supply-side for the expression of anti-Flemish attitudes. In future research, it would be interesting to investigate this region as well.

To conclude, prejudices are clearly interrelated, but not perfectly. Observed prejudices have a clear target-specific component and have different political consequences. Accordingly, target-specific prejudices cannot simply be reduced to a prejudiced personality and must be studied within the particular intergroup context that surrounds its development. The structure of prejudice is, however, not context-specific, at least in Belgium. The different evolutions and experiences in Flanders and Wallonia with regard to the history of immigration, the linguistic and autonomy conflict, the separate party system, and political discourse have activated different social norms with regard to prejudice expressions, but these social norms clearly did not influence how particular prejudices are interrelated. Walloons and Flemings organize their thinking about outgroups in a similar way. The intergroup context, however, does play an important moderating role in the translation of prejudices to party preferences. There is a clear asymmetry between both regions: While anti-immigrant prejudice defines party choice in Flanders and Wallonia, regional prejudice is only relevant for intended political behavior in Flanders. These relationships were group-specific and could not be explained by a generalized prejudice personality. The regional context in Belgium thus matters for the political consequences of prejudice, but not for its structure.

## Additional Files

The additional files for this article can be found as follows:

10.5334/pb.335.s1 Appendix A Multigroup confirmatory factor analysis of the structure of prejudice.

10.5334/pb.335.s2 Appendix B Standardized multivariate regressions of structure of prejudice and party preference.

10.5334/pb.335.s3 Appendix C Standardized univariate regressions of structure of prejudice and party preference controlled for average PTV score.

## Footnotes

We refer to this type of context-specific prejudice as “atypical” because of the particular nature of the structure of the Belgian federation. The citizens we are studying live together in the same country, but apart in their separate language regions (leaving Brussels aside) (in contrast to settings where groups speaking different languages live together, e.g. Quebec). This language-based separation in two regions has led to what we believe is a very specific type of regional prejudice, with its particular stereotypes unique for the Belgian context.

Both structures overlap in Brussels, where both language communities live together. For reasons of simplicity, in this article we focus on the two main regions of Belgium only, i.e., Flanders and Wallonia. That way, we examine attitudes in two clearly opposing groups, that are not only different regions but also represent different language communities. The third language community is the German-speaking one. The community has the same responsibilities as the French- and Dutch-speaking community, but is very small and does not play a key role in Belgian intergroup relations (Deschouwer, 2009). Therefore, this community was not included in the study.

*FDF* (Fédéralistes Démocrates Francophones), currently (since November 2015) *DéFi* (Démocrate Fédéraliste Indépendant) is an outlier in this respect. *FDF/DéFi* is a party focusing mainly on the French language and on the rights of Francophones in the Brussels region. The party can thus be labeled “French regionalist,” but it should not be seen as the regionalist francophone counterpart of *N-VA*: *FDF/Défi* does not strive for independence for Brussels or the Francophone part of the country.

There are no relevant political parties advocating for Walloon regionalism. Note, however, that Walloon regionalism is traditionally leftist (Billiet, Jaspaert & Swyngedouw, 2012), which is another interesting asymmetry between regionalism in both parts of the country.

The far-left *PVDA*, the Green *Groen*, the socialist *Sp.a*, the Christian-democrats *CD&V*, the Liberal *Open VLD*, the Flemish-Nationalist *N-VA*, and the extreme-right *Vlaams Belang*. The very small party *LDD* (Libertarian, Direct and Democratic) was not included. *LDD* was electorally successfully for a short period but was clearly losing in the polls. In the context of the 2014 elections, *LDD* presented electoral lists in only one province (West-Flanders).

In 2014, the party obtained 4.9% of the votes in the Walloon parliament. Its non-inclusion in the questionnaire and in the analyses is therefore a limitation of the current study.

The far-left *PTB*, the Green *Ecolo*, the socialist *PS*, the Christian-democrats *CDH*, the Liberal *MR*, the French Regionalist *FDF*, and the extreme-right *Front National*.

We also fitted a higher-order two-factor model with prejudices toward immigrants and the other regional group loading on the first factor and prejudices toward homosexuals and Jews on the second factor, and a second-order generalized prejudice factor. This model had good fit for the full Belgian sample (χ² = 8.060, df = 3, RMSEA = .035, CFI = .993, TLI = .986), but in the multiple-group design identification problems made this model too complex and uninterpretable. The results do point in the direction of a multidimensional solution to the structure of prejudice, which is further discussed in the Discussion.

In terms of statistical significance, the metric invariance model did not meet the stringent criteria of *p* > .05 for the chi-square difference and Δ < .01 for the difference in CFI values. However, because the model fit differences and the modification indices were only small, we decided to keep the interpretable and parsimonious full metric invariance model. Freeing the factor loading of the item with the highest modification index would have resulted in freeing “positive feelings toward the other regional group”: The factor loading was slightly higher in the Flemish sample than in the Walloon sample. This means that for the Flemings attitudes toward the Walloons are more important in defining their generalized prejudice disposition, than attitudes toward the Flemings are decisive for the Walloons.

We additionally tested models that correct for the tendency to answer with a high propensity to vote for each party. Although the effect sizes became smaller, the conclusions remained the same. Results are in Appendix C.
