# Supplementary material for: The Structure of Prejudice and Its Relation to Party Preferences in Belgium: Flanders and Wallonia Compared
Source: Psychol Belg. 2017 Nov 21;57(3):52–74. doi: 10.5334/pb.335 (PMC6194513; doi:10.5334/pb.335)
Supplement: Appendix A — Multigroup confirmatory factor analysis of the structure of prejudice. [file pb-57-3-335-s1.pdf]

## Appendix A − Multigroup confirmatory factor analysis of the structure of prejudice

| | $\chi^2$ (df) | Model comparison | $\Delta\chi^2$ *p*-value | RMSEA | CFI | TLI |
|---|---|---|---|---|---|---|
| One-group Belgians (*N* = 1392) | | | | | | |
| (1a) One-factor GP | 25.176 (2) | | | .091 | .969 | .906 |
| (1b) One-factor GP + res. cov. immigrant & other regional group | 3.135 (1) | 1a versus 1b | < .001 | .039 | .997 | .983 |
| One-group Flemings (*N* = 768) | | | | | | |
| (2a) One-factor GP | 16.270 (2) | | | .096 | .966 | .898 |
| (2b) One-factor GP + res. cov. immigrant & other regional group | 3.848 (1) | 2a versus 2b | .007 | .061 | .993 | .959 |
| One-group Walloons (*N* = 624) | | | | | | |
| (3a) One-factor GP | 8.366 (2) | | | .071 | .981 | .944 |
| (3b) One-factor GP + res. cov. immigrant & other regional group | 2.097 (1) | 3a versus 3b | .040 | .042 | .997 | .981 |
| Two-group Flemings versus Walloons | | | | | | |
| (4) Configural Invariance | 5.977 (2) | | | .053 | .995 | .969 |
| (5) Equal factor loadings | 18.199 (5) | 4 versus 5 | .029 | .062 | .983 | .959 |
| (6) Equal factor loadings + equal res. cov | 22.234 (6) | 5 versus 6 | .099 | .062 | .979 | .958 |
| (7a) Equal intercepts + equal factor loadings + equal res. cov | 65.933 (9) | 6 versus 7a | < .001 | .095 | .926 | .901 |
| (7b) Model 7a + free intercept homosexuals | 37.366 (8) | 6 versus 7b | < .001 | .073 | .962 | .942 |
| (7c) Model 7b + free intercept immigrants | 23.659 (7) | 6 versus 7c | .238 | .058 | .978 | .963 |

*Note*. Results of multigroup analysis via MLR estimation. Res. cov. = Residual covariance; GP = Generalized prejudice.
