# Supplementary material for: The Structure of Prejudice and Its Relation to Party Preferences in Belgium: Flanders and Wallonia Compared
Source: Psychol Belg. 2017 Nov 21;57(3):52–74. doi: 10.5334/pb.335 (PMC6194513; doi:10.5334/pb.335)
Supplement: Appendix B — Standardized multivariate regressions of structure of prejudice and party preference. [file pb-57-3-335-s2.pdf]

Appendix B – Standardized multivariate regressions of structure of prejudice and party preference

FLANDERS	Far-left		Greens		Socialists		Christian-Democrats		Liberal		Flemish-Nationalist		Extreme-right	
Negative feelings toward **immigrants**	−.141***	−.095	−.331***	−.253***	−.198***	−.100	−.193***	−.152*	−.098*	−.031	.116**	.186**	.368***	.368***
Generalized prejudice		−.006		−.114		−.141		−.058		−.097		−.104		−.034
Gender (ref. male)	.054	.049	.101**	.092**	.089*	.077*	−.046	−.050	.007	.000	−.084*	−.092*	−.057	−.057
Age	−.080	−.084	−.032	−.040	−.074	−.083	−.023	−.027	−.077	−.084	−.037	−.044	−.253***	−.253***
Education	−.103*	−.105*	.146***	.142***	−.045	−.051	.041	.038	.144***	.140***	.051	.048	−.128**	−.128**
SES (ref. manual worker)														
Self-employed	−.024	−.021	−.012	−.007	−.086	−.080	.006	.008	.224***	.227***	.039	.042	−.077	−.077
Non-Manual worker	.091	.090	.165**	.163**	.076	.074	.041	.040	.126	.124	−.003	−.007	−.143*	−.143*
Non-active	.028	.030	.098	.103	.046	.051	.065	.066	.095	.098	.046	.049	−.022	−.021
Religious practice (ref. Catholic non-practicing)														
Non-religious	.152***	.153***	.068	.070	.135***	.137***	−.324***	−.323***	−.110**	−.109**	−.105**	−.104**	−.068*	−.069*
Catholic practicing	−.016	−.012	.062	.069	−.051	−.042	.212***	.217***	−.067	−.060	−.097*	−.090*	−.059	−.059
Other	.120**	.123**	.090**	.095***	.117***	.124***	−.070*	−.067*	−.013	−.009	−.150***	−.146***	−.061*	−.061*
R^2	.081	.084	.186	.190	.110	.118	.214	.217	.098	.102	.056	.062	.186	.187
	Far–left		Greens		Socialists		Christian-Democrats		Liberal		Flemish-Nationalist		Extreme-right	
Negative feelings toward **regional Other**	−.114**	−.014	−.261***	−.055	−.224***	−.191**	−.197***	−.183**	−.123**	−.107	.116**	.230**	.196***	−.062
Generalized prejudice		−.137		−.282***		−.044		−.019		−.021		−.157		.352***
Gender (ref. male)	.045	.043	.080*	.075*	.069	.068	−.063	−.063	−.004	−.004	−.075*	−.078*	−.043	−.035
Age	−.101*	−.093	−.082	−.066	−.110*	−.108*	−.057	−.057	−.098	−.097	−.018	−.008	−.206***	−.226***
Education	−.095*	−.101*	.163***	.152***	−.046	−.049	.042	.041	.139***	.138***	.047	.042	−.160***	−.146***
SES (ref. manual worker)														
Self-employed	−.012	−.014	.014	.011	−.074	−.075	.018	.018	.228***	.228***	.031	.029	−.113*	−.108*
Non-Manual worker	.105	.096	.199***	.181***	.086	.083	.057	.055	.131*	.129*	−.010	−.022	−.191*	−.167*
Non-active	.046	.040	.141*	.129*	.065	.063	.088	.086	.104	.103	.031	.027	−.075	−.061
Religious practice (ref. Catholic non-practicing)														
Non-religious	.161***	.156**	.089*	.080*	.148***	.146***	−.311***	−.312***	−.104**	−.104**	−.113**	−.117**	−.094**	−.081*
Catholic practicing	−.004	−.005	.089*	.088*	−.032	−.032	.231***	.231***	−.056	−.056	−.107*	−.107*	−.085*	−.086*
Other	.125**	.128**	.101***	.110***	.121***	.122***	−.065*	−.065*	−.012	−.012	−.154***	−.150***	−.081*	−.089**
R^2	.080	.086	.168	.191	.126	.126	.212	.212	.105	.105	.057	.067	.118	.171

* p < .05, ** p < .01, *** p < .001

**WALLONIA**	**Far-Left**		**Greens**		**Socialists**		**Christian-Democrats**		**Liberal**		**Regionalists**		**Extreme-right**	
Negative feelings toward **immigrants**	−.108*	−.157**	−.307***	−.320***	−.340***	−.330***	−.170***	−.189***	.174***	.180***	.062	.058	.368***	.387***
Generalized prejudice		.079		.009		−.016		.060		−.050		.013		−.076
Gender (ref. male)	−.006	−.011	.066	.056	.046	.058	−.087	−.060	−.021	−.027	−.095*	−.086*	−.057	−.072
Age	−.186***	−.187***	−.135*	−.115*	.047	.053	−.050	−.034	−.028	−.003	−.085	−.072	−.167***	−.167***
Education	−.041	−.060	.197***	.197***	−.065	−.082	.154***	.167***	.209***	.203***	.105	.122*	.023	−.009
SES (ref. manual worker)														
Self-employed	−.149**	−.110*	−.042	−.045	−.086	−.059	.048	.053	.166**	.135**	.045	.044	−.025	−.017
Non-Manual worker	−.217**	−.148	.037	.063	−.033	.019	.101	.111	.102	.079	.091	.065	−.112	−.126
Non-active	−.025	.012	.131*	.135*	.025	.043	.093	.090	.043	.022	.103	.083	−.107	−.116
Religious practice (ref. Catholic non-practicing)														
Non-religious	.091*	.099*	−.021	−.018	−.023	−.011	−.301***	−.291***	−.111*	−.132**	−.109*	−.104*	−.031	−.019
Catholic practicing	−.008	−.007	.029	.043	−.118**	−.109*	.145**	.177***	.106*	.084	.054	.040	−.061	−.049
Other	.068	.061	−.015	−.026	−.015	−.032	−.052	−.052	−.105**	−.115***	−.049	−.052	−.035	−.031
R^2	.087	.083	.206	.170	.122	.148	.196	.199	.124	.134	.044	.047	.176	.178
	Far-Left		Greens		Socialists		Christian-Democrats		Liberal		Regionalists		Extreme-right	
Negative feelings toward **regional Other**	−.014	−.008	−.053	.062	−.095*	.031	.005	.044	−.042	−.115	.031	.009	−.034	−.179**
Generalized prejudice		−.012		−.234**		−.240**		−.086		.134		.047		.280***
Gender (ref. male)	.005	−.015	.074	.040	.062	.047	−.088*	−.069	−.024	−.012	−.098*	−.085*	−.070	−.045
Age	−.180***	−.179***	−.137**	−.084	.039	.082	−.053	−.015	−.011	−.031	−.077	−.075	−.176***	−.214***
Education	−.004	−.034	.282***	.248***	.019	−.024	.209***	.198***	.153***	.172***	.097	.114*	−.103*	−.074
SES (ref. manual worker)														
Self-employed	−.155**	−.124*	−.059	−.075	−.097	−.088	.042	.036	.190***	.153**	.053	.049	.003	.021
Non-Manual worker	−.221**	−.165*	.025	.026	−.014	−.016	.098	.089	.129	.097	.105	.071	−.093	−.083
Non-active	−.026	.001	.115	.106	.021	.017	.093	.073	.068	.037	.118	.088	−.081	−.078
Religious practice (ref. Catholic non-practicing)														
Non-religious	.099*	.108*	.008	−.003	.003	.004	−.290***	−.281***	−.126**	−.139***	−.121**	−.106*	−.061	−.036
Catholic practicing	−.006	.005	.056	.063	−.106*	−.083	.169***	.189***	.091	.071	.046	.037	−.090**	−.073*
Other	.077	.073	.013	−.001	.015	−.005	−.037	−.037	−.117***	−.129***	−.055	−.056	−.066	−.061
R^2	.075	.065	.124	.126	.032	.076	.174	.184	.108	.117	.044	.044	.059	.120

* $p < .05$, ** $p < .01$, *** $p < .001$.
