# Supplementary material for: The Structure of Prejudice and Its Relation to Party Preferences in Belgium: Flanders and Wallonia Compared
Source: Psychol Belg. 2017 Nov 21;57(3):52–74. doi: 10.5334/pb.335 (PMC6194513; doi:10.5334/pb.335)
Supplement: Appendix C — Standardized univariate regressions of structure of prejudice and party preference controlled for average PTV score. [file pb-57-3-335-s3.pdf]

Appendix C – Standardized univariate regressions of structure of prejudice and party preference controlled for average PTV score

FLANDERS	Far-Left	Greens	Socialists	Christian-Democrats	Liberals	Flemish-nationalists	Extreme-right
Negative feelings toward **immigrants**	−.076	−.215***	−.075	−.130*	−.009	.166***	.343***
Generalized prejudice	.028	−.017	−.040	.002	.000	−.014	.030
PTV mean	.610***	.619***	.569***	.438***	.438***	.438***	.356***
Negative feelings toward **regional Other**	.041	−.007	−.134*	−.134*	−.058	.281***	−.022
Generalized prejudice	−.077	−.198**	.022	.016	.047	−.140**	.358***
PTV mean	.611***	.619***	.565***	.437***	.539***	.445***	.359***
**WALLONIA**	**Far-Left**	**Greens**	**Socialists**	**Christian-Democrats**	**Liberals**	**Regionalists**	**Extreme-right**
Negative feelings toward **immigrants**	−.088*	−.231***	−.269***	−.115**	.239***	.130**	.414***
Generalized prejudice	.072	.009	−.021	.051	−.060	.008	−.065
PTV mean	.562***	.656***	.513***	.637***	.435***	.661***	.427***
Negative feelings toward **regional Other**	−.002	.077	.020	.052	−.073	.050	−.138*
Generalized prejudice	.022	−.176***	−.186**	−.048	.121*	.050	.274***
PTV mean	.568***	.671***	.530***	.642***	.411***	.656***	.389***

Note. PTV mean is calculated as the mean across all PTVs for each party per region. Following control variables were included: gender, age, education, SES, and religious practice. Contrary to the results in Appendix B, the entries are based on univariate regression results per political party. This was necessary to avoid perfect linearity between the PTV mean score and the dependent variables. As a consequence, these results are not directly comparable with the multivariate results in Appendix B.

* $p < .05$, ** $p < .01$, *** $p < .001$.
